# A year-long camera-trap dataset for assessing spatial occurrence and diel activity of sympatric ungulates in South Korea

**DOI:** 10.3897/BDJ.14.e191556

**Published:** 2026-07-10

**Authors:** Jong-Hoon Jeon, Anya Lim

**Affiliations:** 1 Research Center for Endangered Species, National Institute of Ecology, Yeongyang-gun, Gyeongsangbuk-do, Republic of Korea Research Center for Endangered Species, National Institute of Ecology Yeongyang-gun, Gyeongsangbuk-do Republic of Korea

**Keywords:** camera trapping, detection probability, diel activity, occupancy modelling, protected species, South Korea, temporal overlap, ungulates

## Abstract

**Background:**

Understanding how ecologically similar species share space and time is central to community ecology and can provide information for conservation and management in multi-species systems. Camera-trap data provide a practical means of describing spatial co-occurrence and diel activity overlap amongst sympatric mammals, particularly where direct mechanistic inference is not possible from observational data alone. In human-dominated landscapes, standardised, open-access occurrence data are critical for balancing the conservation of endangered species with the management of over-abundant, conflict-prone wildlife.

**New information:**

In this study, we present a curated, year-long camera-trap dataset for four sympatric ungulates in South Korea, based on monitoring conducted in a temperate forest landscape in Uljin County, together with station-level covariates and harmonised effort information derived from a camera operation log. We provide example analyses illustrating: (i) spatial occurrence patterns using species-wise occupancy models and (ii) diel activity and temporal overlap using non-parametric activity analyses. For the temporal example, overlap is summarised using local clock time (Asia/Seoul, UTC+09:00) and complemented by a photoperiod-adjusted (solar-time) sensitivity analysis, based on sunrise and sunset times calculated for site-level representative coordinates (one point per site; UJ1 and UJ2). Diel-period summaries are additionally reported as rate-normalised detections (detections per 100 hours) using civil-twilight definitions to account for unequal period durations. The released dataset contains 4,623 independent detection events for the four ungulate species (long-tailed goral: 2,317; water deer: 814; Siberian roe deer: 808; wild boar: 684) recorded at 82 camera stations between April 2022 and May 2023. Survey effort totalled 29,850 functional camera-trap nights (30,410 scheduled nights, including 560 inactive nights from recorded downtime). All released files are listed in the Data resources section and include event-level records, station-level covariates, generalised coordinates and effort summaries to support reproducible reuse.

## Introduction

Quantifying how ecologically similar species share space and time within landscapes is a fundamental topic in community ecology (Hutchinson 1959, HilleRisLambers et al. 2012, Andersen et al. 2020). Overlap amongst ecologically similar species can reflect a combination of shared resource use, behavioural interactions and responses to environmental heterogeneity; however, observational studies typically quantify patterns of overlap and association rather than directly identifying underlying mechanisms (de Boer and Prins 1990, Finke and Snyder 2008, Mori et al. 2020, Zanni et al. 2021, Hiruma et al. 2023).

Standardised observation data are essential for assessing these spatial and temporal associations, especially in regions where multiple species overlap in space and time. Ungulates, as large herbivores, significantly influence ecosystem structure and function through browsing, grazing and nutrient cycling (Latham 1999, Ritchie and Olff 1999, Olff et al. 2002, Pringle et al. 2023). Given their ecological importance, understanding how ungulates share space and time is essential for effective ecosystem management and biodiversity conservation.

In South Korea, five ungulate species are native: the long-tailed goral (*Naemorhedus caudatus*), Siberian musk deer (*Moschus moschiferus*), water deer (*Hydropotes inermis*), Siberian roe deer (*Capreolus pygargus*) and wild boar (*Sus scrofa*) (Kim et al. 2023). However, the Siberian musk deer is currently known from a limited range in Gangwon Province, including areas around the Demilitarized Zone (DMZ), and its small population size and rugged mountainous habitat make field observation and ecological study particularly difficult (Kim et al. 2024). Therefore, our study focused on four ungulate species that were detected in the camera-trap survey: the long-tailed goral, water deer, Siberian roe deer and wild boar.

Generating baseline data on these four species is highly valuable for assessing spatial occurrence and diel activity because of their contrasting conservation and management statuses. The long-tailed goral is a protected species that relies heavily on rugged, forested habitats. Conversely, the water deer and wild boar often occur in human-modified landscapes, where range expansion and overlap with agricultural or peri-urban areas can increase human–wildlife conflict. Although these species overlap broadly in distribution, prior studies report differences in habitat associations and diel activity. The long-tailed goral is primarily crepuscular (Park et al. 2018), while the water deer is active throughout the day with a peak at dusk (Eom et al. 2023). Siberian roe deer display bimodal activity (Mori et al. 2021, Jia et al. 2023, Wang et al. 2024) and wild boar are predominantly nocturnal (Boitani et al. 1994, Russo et al. 1997, Brivio et al. 2017).

However, few studies have jointly quantified spatio-temporal association patterns amongst these sympatric ungulates in South Korea using a standardised sampling design. Previous work has often focused on single species in isolation, leaving limited evidence on how these ungulates overlap in space and time when monitored simultaneously. To address this gap, we deployed a camera-trap network across multiple sites and seasons to generate a year-long dataset for assessing spatial occurrence and diel activity in sympatric ungulates.

Here, we release a curated, year-long camera-trap dataset for four sympatric ungulates in South Korea, together with station-level covariates and harmonised effort derived from a camera operation log. To demonstrate reuse and validate the dataset’s utility, we provide example workflows for: (i) species-wise occupancy modelling and (ii) non-parametric temporal overlap analysis, including a photoperiod-adjusted (solar-time) sensitivity analysis, based on sunrise and sunset times calculated for site-level representative coordinates (one point per site; UJ1 and UJ2).

## General description

### Purpose

The purpose of this data paper is to release a curated, year-long camera-trap dataset from Uljin County, South Korea, for assessing spatial occurrence and diel activity of four sympatric ungulates, and to provide example workflows that facilitate reproducible reuse.

### Additional information

The released dataset comprises 4,623 independent detection events recorded at 82 camera stations between April 2022 and May 2023, representing 29,850 functional camera-trap nights of survey effort (see New information and Data resources for file-level details).

## Project description

### Study area description

**Figure 1.**
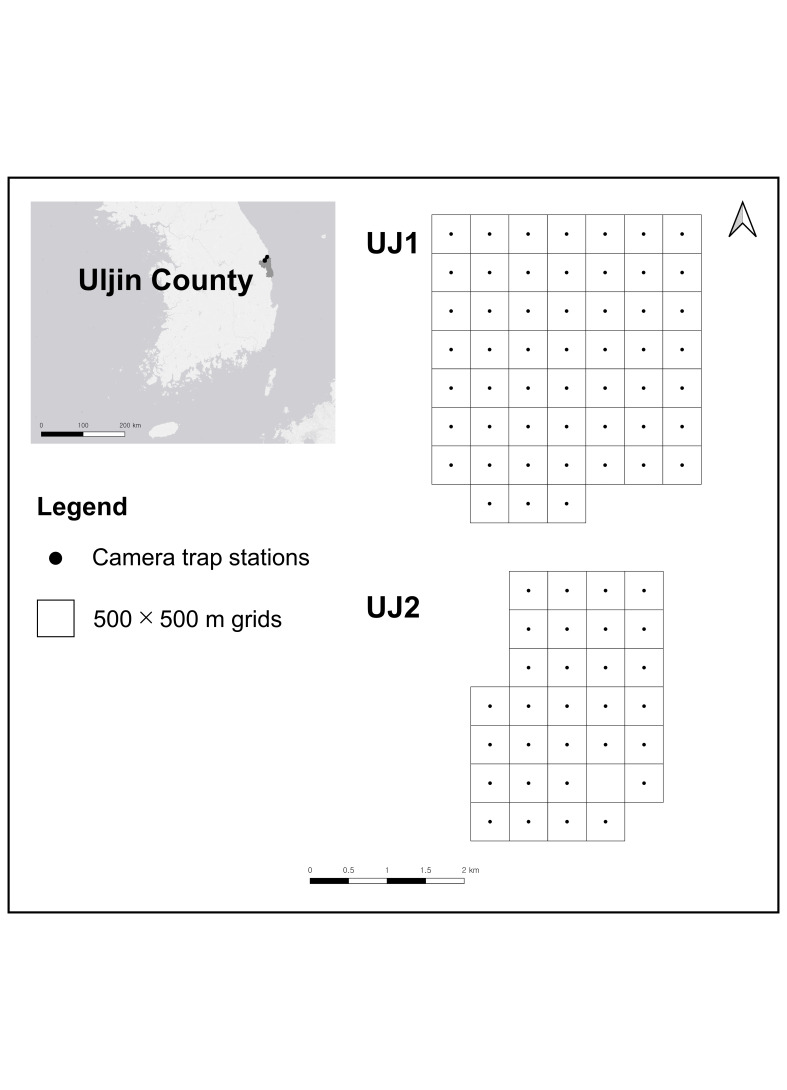
Study area and camera-trap sampling design in Uljin County, South Korea. Camera-trap stations were assigned to 500 × 500 m grid cells in two study sites (UJ1 and UJ2). To ensure the geoprivacy of a protected species, exact station coordinates are withheld; the figure shows only site-level generalised locations (one representative point per site) and the sampling grid layout, not the positions of individual stations. Maps were prepared in QGIS 3.28.7 (QGIS Development Team 2024).

This study was conducted in Uljin County, Gyeongbuk Province, South Korea, within the eastern coastal region of the Korean Peninsula (approximately 36.6–37.1°N, 129.0–129.5°E; Fig. 1). Uljin spans an area of approximately 990 km² along the eastern coast and lies within Nakdongjungmaek, a mountain range characterised by rugged terrain and narrow valleys. Elevations range from sea level to 1,137 m. The region falls within a temperate climate zone, with an average annual temperature of 12.5°C (range: -16.6 to 37.2°C) and an average annual precipitation of 1,225 mm (Korea Meteorological Administration 2024). Two study sites were selected within this region, designated as UJ1 and UJ2. Both sites are dominated by mixed forests of coniferous and broadleaf species, including *Pinus densiflora*, *Quercus variabilis* and *Q. mongolica*. Uljin represents the southernmost range of a viable population of the endangered long-tailed goral (Bragina et al. 2020), making it an important area for conservation-focused research.

### Design description

A total of 82 infrared camera traps (Reconyx HP2X Hyperfire 2, Reconyx Inc., USA) were deployed across the study sites from April 2022 to May 2023 to monitor ungulate populations. The study sites were overlaid with 0.25 km² grid cells (500 × 500 m) and one camera trap was placed within 25 m of each grid centroid where feasible. A total of 52 cameras were deployed in UJ1 and 30 in UJ2 (Fig. 1). Cameras were mounted on trees with a diameter at breast height (DBH) ≥ 20 cm, positioned 80–100 cm above ground and orientated as close to parallel to the ground as possible, with a focal distance of at least 3 m. Cameras were set to operate continuously, 24 h per day, capturing three images per trigger event with no delay between successive detections. To reduce placement bias, cameras were placed along natural animal trails without the use of lures or baits. The stations were maintained across the study period from April 2022 to May 2023, with memory cards and lithium batteries replaced as necessary.

## Sampling methods

### Sampling description

For each camera-trap image, we recorded species identity and timestamp information (date and time). To reduce pseudoreplication, detections of the same species at the same camera location were treated as independent only if separated by at least 30 minutes (Linkie and Ridout 2011, Chen et al. 2022). The released event-level dataset reports these 30-minute independent events and their timestamps in local standard time (Asia/Seoul, UTC+09:00). The example workflows in this data paper (occupancy and activity-overlap workflows) use the released independent-event records; users may adapt the time-to-independence threshold for alternative reuse scenarios if needed, noting that inappropriate choices can bias activity estimates (Peral et al. 2022).

We released station-level covariates in the public site-covariates table (Table 1), including topographic descriptors derived from a digital elevation model (elevation, slope, aspect and hillshade) and an anthropogenic index defined as the frequency of independent human detections at each station (frequency_of_people; using the same 30-minute independence rule). For spatial geoprivacy and protected-species safeguarding, exact camera-station coordinates and fine-scale locality descriptors are withheld from the public dataset. Public records provide only generalised, site-level representative coordinates (UJ1 and UJ2 centroids) consistent with Fig. 1. Topographic covariates (elevation, slope, aspect and hillshade) were derived from NASADEM HGT v001 (1 arc-second resolution, approximately 30 m) tiles. The DEM tiles were mosaicked and projected to WGS 84 / UTM Zone 52N (EPSG:32652) in QGIS and terrain derivatives were computed using GDAL terrain analysis tools (slope in degrees; aspect in degrees from 0 to 360). Hillshade values (0–255) were calculated with standard illumination parameters (azimuth 315°, altitude 45°, z-factor 1). Raster values were then extracted to each camera-trap station point to populate the station-level covariates table. Detection-level covariates used in the example occupancy workflow include season and detection effort. Seasons were defined by month as spring (Mar–May), summer (Jun–Aug), autumn (Sep–Nov) and winter (Dec–Feb). Detection effort was quantified as the number of functional camera-trap nights per station and season using the released camera operation log (deployment start/end dates and recorded downtime intervals).

### Quality control

Quality control procedures followed the event-level annotation workflow and the independence rule described above; effort and downtime were harmonised using the released camera operation log.

- Species identification review: All images were annotated for species identity and timestamp, followed by a secondary review to resolve uncertain labels. Records that could not be confidently assigned to species were conservatively assigned to a higher taxonomic level (e.g. genus) or were excluded from the released focal-species event dataset.
- Taxonomic standardisation: Each record includes an accepted scientific name and the corresponding English common name, harmonised to the GBIF Backbone Taxonomy with synonym resolution and applied consistently across all released tables (events, covariates and example-analysis inputs).
- Human privacy and site protection: Images containing people were screened and used solely to compute an anthropogenic disturbance index (frequency of independent human events, defined using the 30-minute independence rule; camera-trap operators and maintenance events were excluded). No personally identifiable images were published or shared. To mitigate location-disclosure risks for protected species, specifically the endangered long-tailed goral, exact camera-station coordinates and fine-scale locality descriptors are withheld from the public dataset. Public records provide only site-level generalised coordinates (one representative point per site; UJ1 and UJ2) consistent with Fig. 1.
- Data integrity checks: We conducted automated QA checks for duplicate event records, timestamp parsing and validity checks (date/time format and time-zone consistency) and consistency against deployment windows and downtime logs derived from the camera operation log; QA summary outputs are provided in the release package.

## Geographic coverage

### Description

The study was conducted in Uljin County, Gyeongbuk Province, South Korea (Fig. 1), at two temperate-forest study sites (UJ1 and UJ2). Location information is reported at a site-level generalised resolution to ensure the geoprivacy of protected species.

### Coordinates

36°6′ and 37°1′ Latitude; 129°0′ and 129°5′ Longitude.

## Taxonomic coverage

### Description

The dataset includes four sympatric ungulates recorded in camera-trap surveys in Uljin County, South Korea: long-tailed goral (*Naemorhedus caudatus*), water deer (*Hydropotes inermis*), Siberian roe deer (*Capreolus pygargus*) and wild boar (*Sus scrofa*). Species labels in the event records and example analyses use English common names and each record includes the accepted scientific name in the scientificName field, harmonised to the GBIF Backbone Taxonomy, to support reuse and interoperability.

## Temporal coverage

### Notes

Camera trapping was conducted from 28 April 2022 to 31 May 2023, according to the released camera operation log. The released focal-ungulate independent-event records span 1 May 2022 to 22 May 2023. Event timestamps are reported in local standard time (Asia/Seoul, UTC+09:00) using the 30-minute independent-event definition described above.

## Usage licence

### Usage licence

Creative Commons Public Domain Waiver (CC-Zero)

### IP rights notes

For journal metadata purposes, the user licence is indicated as CC0. The EcoBank-archived release itself is made available under the Korea Open Government License (KOGL) Type 1 (Attribution), which permits use, redistribution and adaptation provided that appropriate attribution is given.

## Data resources

### Data package title

Camera-trap event records and site-level coordinates with station covariates for sympatric ungulates in Uljin, South Korea (public dataset v1.0, 2026-03-05)

### Resource link

https://doi.org/10.22756/ETC.20260000001022

### Number of data sets

1

### Data set 1.

#### Data set name

Camera-trap event records and site covariates for sympatric ungulates in Uljin, South Korea

#### Data format

CSV

#### Character set

UTF-8

#### Download URL

https://doi.org/10.22756/ETC.20260000001022

#### Description

This public data package archived in the NIE EcoBank repository contains event-level camera-trap records and associated station-level covariates for four sympatric ungulate species recorded in Uljin, South Korea. The released archive includes: (i) a camera operation log (01_core_data/camera_operation_log.csv) with operation windows and downtime intervals; (ii) event-level camera-trap records (01_core_data/cameratrap_event_records.csv) with Station, Species, scientificName, Date and Time; (iii) station-level covariates and generalised site-level representative coordinates (01_core_data/site_covariates_public.csv), including topographic variables, frequency_of_people and coordinateUncertaintyInMeters; (iv) a station × season effort summary (01_core_data/station_season_effort.csv) with scheduled, inactive and functional nights; and (v) QA summary tables (02_quality_checks/analysis_input_QA.csv and 02_quality_checks/operation_effort_QA_summary.csv). Documentation is provided in 03_documentation/README_EcoBank.txt and 03_documentation/MANIFEST.tsv. Exact station coordinates are withheld; public coordinates are generalised site-level representative coordinates with coordinateUncertaintyInMeters = 10000. The EcoBank archive has been updated as Version 1.1.

**Data set 1.** 

Column label	Column description
Station	Unified camera-station ID; site is derivable from the prefix (UJ1 or UJ2).
Species	English common-name label used in the dataset (Goral, Water deer, Roe deer, Wild boar).
scientificName	Accepted scientific name corresponding to Species.
Date	Date of the independent detection event (YYYY-MM-DD).
Time	Time of the independent detection event (HH:MM:SS).

## Additional information

### Example analyses

To illustrate potential reuse of the dataset for assessing spatial occurrence and diel activity, we provide an example occupancy modelling workflow for the four ungulates using single-species occupancy models (MacKenzie et al. 2002), with one model fitted for each species. Detection histories were constructed from the released independent-event records by defining a detection for a station–season when at least one independent event occurred for a given species. Species labels follow the released English common-name field (*Species*), with accepted scientific names provided for each record in the scientificName field for interoperability.

Sampling occasions were defined at the station–season level (spring: Mar–May; summer: Jun–Aug; autumn: Sep–Nov; winter: Dec–Feb) and detection effort was included as the number of functional camera-trap nights in each station–season derived from the camera operation log. The detection component included season indicators and log-transformed effort, while the occupancy component used released station covariates (elevation, slope, frequency_of_people and aspect represented by sine/cosine terms).

We fitted three candidate occupancy models for each species (M0–M2), where M0 included an intercept-only occupancy component, M1 included elevation, slope and frequency_of_people and M2 additionally included aspect (sine and cosine). Models were fitted in R using the *unmarked* package (Fiske and Chandler 2011) and compared using AICc (Arnold 2010). Parameter estimates are reported with 95% confidence intervals from the fitted models to illustrate a reusable workflow rather than for causal inference.

As some long-tailed goral occupancy parameters appeared unstable in the full candidate set, we additionally conducted a reduced-model sensitivity check for this species. This check used progressively simpler occupancy structures while retaining the same station–season detection histories and detection model and was intended to assess parameter stability within the example workflow.

To describe diel activity patterns, we estimated continuous activity density curves using kernel density estimation of time-of-detection data. Activity curves were summarised using local clock time (Asia/Seoul, UTC+09:00) pooled across seasons to provide an overall description of detection timing. We additionally classified detections into diel periods defined relative to sunrise and sunset using civil twilight times computed for the site-level representative coordinates (one point per site for UJ1 and UJ2): daytime (sunrise–sunset), crepuscular (dawn to sunrise and sunset to dusk) and night-time (dusk–next dawn). As crepuscular windows are shorter than daytime and night-time, diel-period summaries are reported as rate-normalised detections (detections per 100 hours).

To quantify pairwise similarity in diel activity, we estimated the coefficient of overlap (Δ) using kernel density estimation of time-of-detection data (Ridout and Linkie 2009, Meredith et al. 2024). Given the sample sizes, we report overlap estimates using the Δ4 estimator (Dhat4) and uncertainty was quantified using non-parametric bootstrap confidence intervals (B = 1000). Overlap was calculated using local clock time and complemented by a photoperiod-adjusted (solar-time) sensitivity analysis in which detection times were rescaled relative to sunrise and sunset computed at the site-level representative coordinates, such that daytime and nighttime each span 12 hours (06:00–18:00 h and 18:00–06:00 h, respectively). Pairwise density plots for all species pairs are provided in Suppl. material 1, photoperiod-adjusted (solar-time) activity curves and overlap estimates in Suppl. material 2 and diel-period rate-normalised summaries (detections per 100 hours) in Suppl. material 3.

### Example results

**Figure 2.**
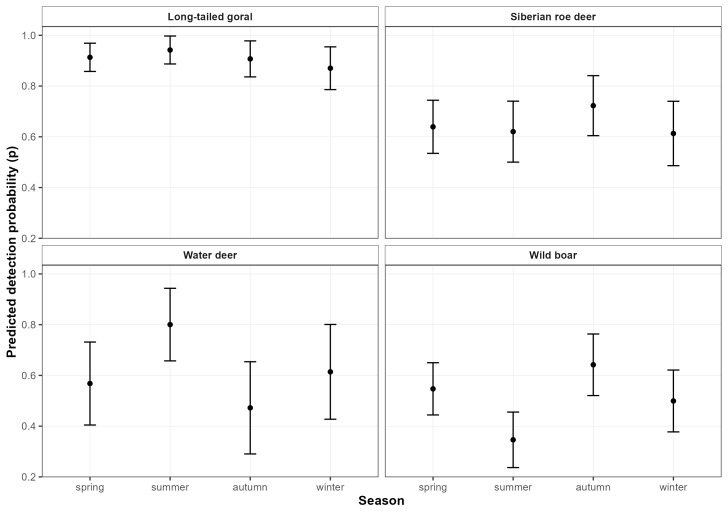
Predicted detection probability (p) by season for four ungulate species. Predictions are derived from the best-supported model for each species (Table 2) and evaluated at the mean survey effort (log_effort_z = 0). Error bars indicate 95% confidence intervals.

**Figure 3.**
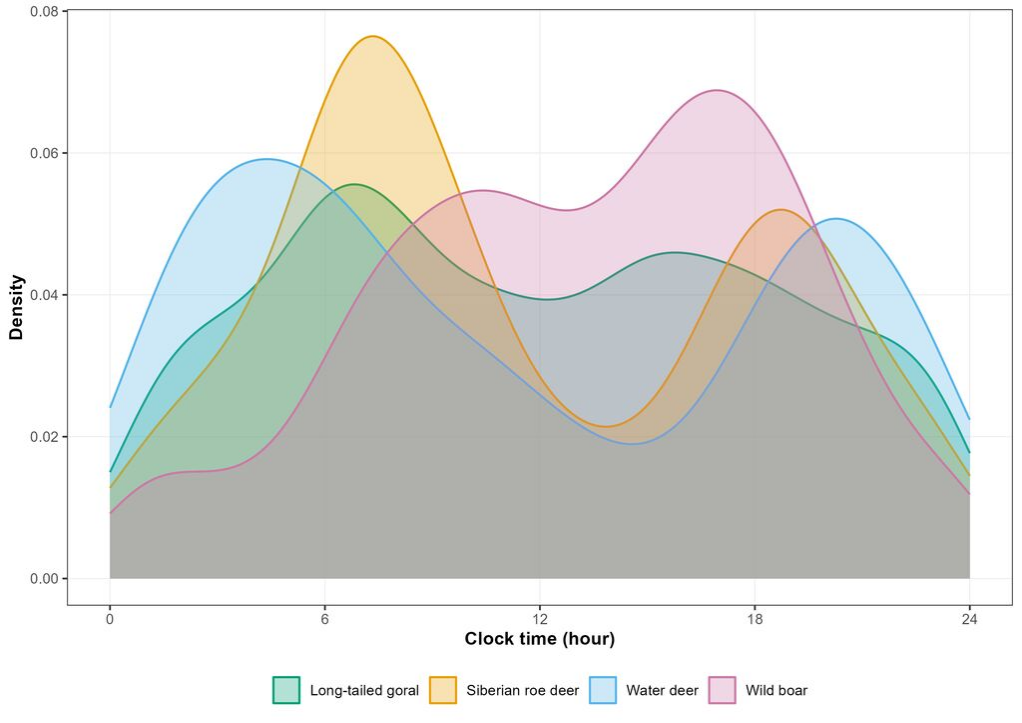
Diel activity density curves, based on local clock time for four ungulate species. Curves show kernel density estimates of detection times recorded in local standard time (Asia/Seoul, UTC+09:00); species colours were kept consistent to support cross-figure comparisons.

Example outputs from the released dataset are summarised in Tables 2–4 and Figs 2–3. For the occupancy workflow, we fitted species-wise occupancy models using station-level covariates in the occupancy component (ψ) and season plus survey effort in the detection component (p) and selected the best-supported model for each species using AICc (Table 2). Species are reported using English common names, with accepted scientific names provided in the scientificName field.

Predicted detection probabilities, evaluated at the mean survey effort, varied amongst species and seasons (Fig. 2). Parameter estimates from the best-supported model for each species are reported to illustrate a reusable and reproducible workflow rather than for causal inference (Table 3). For long-tailed goral, the reduced-model sensitivity check indicated that, although the more parameter-rich occupancy structure remained the best-supported model by AICc, several occupancy coefficients remained inflated and imprecise, suggesting weak parameter identification. Therefore, this example should be interpreted as a workflow demonstration rather than as strong evidence for specific covariate effects. Some parameters may, therefore, be weakly identified for certain species–covariate combinations and the estimates should not be interpreted causally.

Using pooled clock-time detection timestamps (Asia/Seoul, UTC+09:00), pairwise overlap ranged from Δ = 0.653 to 0.840 (Table 4; Fig. 3; Suppl. material 1). The highest overlap was between long-tailed goral and Siberian roe deer (Δ = 0.840; 95% CI: 0.803–0.872), while the lowest was between water deer and wild boar (Δ = 0.653; 95% CI: 0.609–0.692). Photoperiod-adjusted (solar-time) overlap estimates were similar (Δ = 0.633–0.819; Suppl. material 2) and diel-period summaries are also reported as rate-normalised detections (detections per 100 hours; Suppl. material 3).

### Interpretation and reuse notes

This data paper provides curated event-level records, station-level covariates and harmonised effort information to support reuse in studies of occurrence, detectability and diel activity. Users may adapt the released covariate set and modelling choices (e.g. alternative temporal aggregation, different covariate transformations or sensitivity analyses using alternative independence thresholds) according to specific hypotheses, while ensuring that any changes are fully documented for reproducibility. For spatial privacy and protected-species safeguarding, exact station coordinates are withheld from the public release; users should treat location information as site-level generalised and avoid attempts to infer fine-scale station positions.

The example analyses are intended to illustrate reproducible workflows for describing patterns of occurrence, detectability and temporal overlap. They should not be interpreted as evidence of causal effects or direct tests of ecological mechanisms. Occupancy–covariate relationships should be viewed as statistical associations within the sampled landscape and temporal overlap estimates indicate similarity in activity patterns rather than behavioural interactions or resource partitioning. As the dataset is observational, the underlying mechanisms cannot be identified from camera-trap records alone. Future studies combining camera trapping with GPS telemetry, dietary analyses, habitat-use measurements or experimental approaches could help clarify these mechanisms.

Siberian musk deer is not represented in this dataset because it does not occur within the Uljin study area and is currently restricted to parts of Gangwon Province, including areas near the DMZ. Therefore, the released archive and example analyses should be interpreted as representing only the four ungulate species detected during the survey, within the geographic context of the dataset, rather than all sympatric ungulates in South Korea.

### Ethics and permits

This research was conducted in compliance with the Wildlife Protection and Management Act of South Korea. Fieldwork permissions and access to national forest areas were granted by the Korea Forest Service (Uljin National Forest Office; permit no. UljinNationalForestOffice-2007, 25 March 2022). As this study relied exclusively on non-invasive camera trapping without the use of lures or baits and involved no animal capture, handling or physical interaction, approval from an Institutional Animal Care and Use Committee (IACUC) was not required.

To minimise behavioural disturbance and support animal welfare, we used infrared flash cameras (Reconyx HP2X) to reduce startle responses and followed the taxon-specific guidelines of the American Society of Mammalogists (Sikes and Animal Care and Use Committee of the American Society of Mammalogists 2016). Regarding human privacy, images containing people were screened and used solely to calculate anthropogenic disturbance frequency; no personally identifiable images were published or shared.

### Declarations

- Funding statement

This work was supported by the Nationwide Survey on the Habitat Status and Conservation Research of Long-tailed Goral project, funded by the Ministry of Climate, Energy and Environment (MCEE) of the Republic of Korea.

- Author contributions

Jong-Hoon Jeon: Investigation (fieldwork), Data curation, Methodology, Writing - original draft, Writing - review and editing.

Anya Lim: Conceptualisation, Supervision, Methodology, Formal analysis, Visualisation, Writing - review and editing.

- Competing interests

The authors declare no competing interests.

- AI-assisted writing statement

We used an AI-assisted writing tool (ChatGPT; OpenAI) solely to support English-language editing and grammatical refinement. The authors reviewed the output and take full responsibility for the final text and all scientific content.

- Data availability

The dataset and accompanying materials are provided as supplementary materials to this article. In addition, the full release is archived in the National Institute of Ecology (NIE) EcoBank data repository under https://doi.org/10.22756/ETC.20260000001022. On the EcoBank landing page, the dataset title is retained as 'public dataset v1.0', while the revised archive is registered there as Version 1.1.

Recommended data citation: National Institute of Ecology. (2026). Camera-trap event records and site covariates for sympatric ungulates in Uljin, South Korea (public dataset v1.0, 2026-03-05) (Version 1.1) [Data set]. https://doi.org/10.22756/ETC.20260000001022.

## Supplementary Material

56238AF0-B04B-5B13-A0CF-C5D7FC4F524E 10.3897/BDJ.14.e191556.suppl1 Supplementary material 1 Pairwise density plots for all species pairs Data type images and table Brief description Pairwise density plots for all species pairs, based on pooled clock-time detection timestamps (Asia/Seoul, UTC+09:00), provided as a ZIP archive containing one PDF of pairwise density plots, one rounded TSV table of pairwise Δ estimates and six pair-specific PNG figures. File: oo_1559452.zip https://binary.pensoft.net/file/1559452 Jong-Hoon Jeon, Anya Lim

ACFFFD72-A1FD-56AB-B94C-C29DCAD57CBD 10.3897/BDJ.14.e191556.suppl2 Supplementary material 2 Photoperiod-adjusted (solar-time) activity curves and overlap estimates Data type images and table Brief description Photoperiod-adjusted (solar-time) activity curves and overlap estimates for sympatric ungulate species, provided as a ZIP archive containing one PDF and one PNG of solar-time activity curves and one CSV table of photoperiod-adjusted overlap estimates for sensitivity comparison with the pooled clock-time analyses in the main text. File: oo_1559453.zip https://binary.pensoft.net/file/1559453 Jong-Hoon Jeon, Anya Lim

04BA6CD2-B818-5CB2-9F6E-ECAF934A6637 10.3897/BDJ.14.e191556.suppl3 Supplementary material 3 Diel-period rate-normalised summaries (detections per 100 hours) Data type image and table Brief description Diel-period summaries reported as rate-normalised detections (detections per 100 hours), provided as a ZIP archive containing one PNG figure and one TSV table supporting the temporal activity interpretation in the main text. File: oo_1559451.zip https://binary.pensoft.net/file/1559451 Jong-Hoon Jeon, Anya Lim

## Figures and Tables

**Table 1.** Description of candidate station-level covariates and site-level representative coordinates released with the camera-trap dataset.

Domain	Variable name (CSV column)	Definition / calculation	Data source	Spatial resolution	Unit / scale	Notes (direction; QC)
Spatial	Station	Camera station identifier (unique code)	Assigned	-	string	Matches event file Station; formatted as UJ1_01–UJ1_52 and UJ2_01–UJ2_30
Spatial	Site	Study site label for each station (UJ1 or UJ2)	Assigned	Site-level	-	Used to group stations for site-level coordinate generalisation
Spatial	utm_x	Generalised UTM easting coordinate (EPSG:32652) for the study site representative point (UJ1 or UJ2)	Derived from internal station coordinates	Site-level (UJ1/UJ2)	m	Generalised site-level representative coordinate (not station-level); identical for all stations within a site; exact station coordinates are withheld
Spatial	utm_y	Generalised UTM northing coordinate (EPSG:32652) for the study site representative point (UJ1 or UJ2)	Derived from internal station coordinates	Site-level (UJ1/UJ2)	m	Generalised site-level representative coordinate (not station-level); identical for all stations within a site; exact station coordinates are withheld
Spatial	decimalLatitude	Generalised latitude (WGS84, EPSG:4326) for the study site representative point (UJ1 or UJ2)	Derived from internal station coordinates	Site-level (UJ1/UJ2)	decimal degrees	Generalised site-level representative coordinate (one point per site; not station-level); identical for all stations within a site; exact station coordinates are withheld
Spatial	decimalLongitude	Generalised longitude (WGS84, EPSG:4326) for the study site representative point (UJ1 or UJ2)	Derived from internal station coordinates	Site-level (UJ1/UJ2)	decimal degrees	Generalised site-level representative coordinate (one point per site; not station-level); identical for all stations within a site; exact station coordinates are withheld
Spatial	coordinateUncertaintyInMeters	Estimated positional uncertainty for the generalised site-level coordinates	Assigned (conservative)	Site-level (UJ1/UJ2)	m	Applies to the site-level representative coordinates; conservative uncertainty to reduce location disclosure risks for protected species; exact station coordinates are withheld
Topography	elevation	Elevation above sea level at station	NASADEM (1 arc-second)	ca. 30 m	m	Derived after mosaicking and projection to EPSG:32652; extracted at station
Topography	slope	Slope at station	Derived from projected DEM (GDAL slope)	ca. 30 m	degrees	Computed using GDAL slope (degrees)
Topography	aspect	Aspect at station (azimuth)	Derived from projected DEM (GDAL aspect)	ca. 30 m	degrees	Computed using GDAL aspect (0–360°; 0/360 = North)
Topography	hillshade	Hillshade index at station	Derived from projected DEM (GDAL hillshade)	ca. 30 m	unitless	Computed using GDAL hillshade (0–255; azimuth 315°, altitude 45°, z-factor 1)
Anthropogenic	frequency_of_people	Frequency of independent human detections at station (30-min rule)	Derived from camera-trap records	Station-level	count	Computed from human detections using the same 30-min independence rule

**Table 2.** Model selection (AICc) for the example occupancy workflow (species-wise models). Note: Candidate occupancy models were M0 (ψ ~ 1), M1 (ψ ~ elevation_z + slope_z + people_z) and M2 (ψ ~ elevation_z + slope_z + people_z + asp_sin_z + asp_cos_z); the detection model was p ~ log_effort_z + season (spring/summer/autumn, with winter as the baseline).

**Species**	**Model**	**AIC**	**AICc**	**deltaAICc**
Long-tailed goral	M2	246.28	250.05	0
Long-tailed goral	M1	256.56	259.06	9.01
Long-tailed goral	M0	261.95	263.07	13.03
Siberian roe deer	M1	439.23	441.73	0
Siberian roe deer	M2	441.82	445.60	3.86
Siberian roe deer	M0	450.15	451.27	9.54
Water deer	M1	255.16	257.66	0
Water deer	M2	254.69	258.46	0.80
Water deer	M0	284.97	286.09	28.43
Wild boar	M1	473.01	475.51	0
Wild boar	M2	473.19	476.96	1.44
Wild boar	M0	480.79	481.91	6.40

**Table 3.** Parameter estimates of the best-supported occupancy models (logit scale). Note: ψ denotes occupancy and p denotes detection. Estimates are shown with SE and Wald 95% CI. Season indicators are relative to winter; log_effort_z is the standardised log of (functional nights + 1).

**Species**	**BestModel**	**Component**	**Term**	**Estimate**	**SE**	**95% CI**
Long-tailed goral	M2	p	Intercept	1.903	0.381	[1.157, 2.650]
Long-tailed goral	M2	p	log_effort_z	0.601	0.172	[0.265, 0.937]
Long-tailed goral	M2	p	is_spring	0.451	0.558	[-0.643, 1.544]
Long-tailed goral	M2	p	is_summer	0.886	0.636	[-0.361, 2.134]
Long-tailed goral	M2	p	is_autumn	0.375	0.567	[-0.736, 1.486]
Long-tailed goral	M2	ψ	Intercept	57.824	173.651	[-282.531, 398.180]
Long-tailed goral	M2	ψ	elevation_z	30.516	274.127	[-506.774, 567.806]
Long-tailed goral	M2	ψ	slope_z	4.224	60.575	[-114.503, 122.950]
Long-tailed goral	M2	ψ	people_z	-1.143	11.353	[-23.395, 21.110]
Long-tailed goral	M2	ψ	asp_sin_z	15.062	111.212	[-202.914, 233.037]
Long-tailed goral	M2	ψ	asp_cos_z	2.749	13.080	[-22.887, 28.386]
Siberian roe deer	M1	p	Intercept	0.461	0.273	[-0.074, 0.996]
Siberian roe deer	M1	p	log_effort_z	0.756	0.167	[0.428, 1.084]
Siberian roe deer	M1	p	is_spring	0.112	0.371	[-0.615, 0.839]
Siberian roe deer	M1	p	is_summer	0.030	0.369	[-0.693, 0.754]
Siberian roe deer	M1	p	is_autumn	0.498	0.394	[-0.275, 1.270]
Siberian roe deer	M1	ψ	Intercept	2.504	0.676	[1.179, 3.829]
Siberian roe deer	M1	ψ	elevation_z	-0.928	0.391	[-1.695, -0.161]
Siberian roe deer	M1	ψ	slope_z	-0.808	0.373	[-1.538, -0.077]
Siberian roe deer	M1	ψ	people_z	1.242	1.181	[-1.073, 3.557]
Water deer	M1	p	Intercept	0.335	0.433	[-0.514, 1.185]
Water deer	M1	p	log_effort_z	0.487	0.155	[0.183, 0.791]
Water deer	M1	p	is_spring	-0.186	0.326	[-0.824, 0.453]
Water deer	M1	p	is_summer	1.234	0.378	[0.493, 1.974]
Water deer	M1	p	is_autumn	-0.580	0.334	[-1.234, 0.074]
Water deer	M1	ψ	Intercept	0.941	0.388	[0.181, 1.702]
Water deer	M1	ψ	elevation_z	-0.554	0.211	[-0.968, -0.140]
Water deer	M1	ψ	slope_z	0.229	0.222	[-0.206, 0.664]
Water deer	M1	ψ	people_z	-0.601	0.331	[-1.250, 0.048]
Wild boar	M1	p	Intercept	0.303	0.245	[-0.177, 0.783]
Wild boar	M1	p	log_effort_z	0.505	0.099	[0.311, 0.700]
Wild boar	M1	p	is_spring	0.195	0.205	[-0.207, 0.597]
Wild boar	M1	p	is_summer	-0.888	0.247	[-1.372, -0.404]
Wild boar	M1	p	is_autumn	0.563	0.266	[0.041, 1.085]
Wild boar	M1	ψ	Intercept	2.311	0.626	[1.084, 3.538]
Wild boar	M1	ψ	elevation_z	-0.543	0.353	[-1.235, 0.149]
Wild boar	M1	ψ	slope_z	-0.647	0.376	[-1.384, 0.090]
Wild boar	M1	ψ	people_z	0.057	0.570	[-1.060, 1.174]

**Table 4.** Pairwise temporal overlap (Δ) in diel activity among four ungulate species, based on local clock time. Overlap coefficients (Δ) were estimated from kernel density estimates of detection times in local clock time (Asia/Seoul, UTC+09:00). The Δ4 estimator (Dhat4) was used for all pairs given the sample sizes and uncertainty was quantified using non-parametric bootstrap confidence intervals (B = 1000).

**Species 1**	**Species 2**	**n1**	**n2**	**Estimator**	**Δ**	**95% CI**
Long-tailed goral	Siberian roe deer	2317	808	Dhat4	0.840	0.803–0.872
Long-tailed goral	Water deer	2317	814	Dhat4	0.811	0.774–0.842
Long-tailed goral	Wild boar	2317	684	Dhat4	0.810	0.770–0.841
Siberian roe deer	Water deer	808	814	Dhat4	0.799	0.746–0.829
Siberian roe deer	Wild boar	808	684	Dhat4	0.757	0.706–0.789
Water deer	Wild boar	814	684	Dhat4	0.653	0.609–0.692
