# Supplementary figures and images for: A year-long camera-trap dataset for assessing spatial occurrence and diel activity of sympatric ungulates in South Korea
Source: Biodivers Data J. 2026 Jul 10;14:e191556. doi: 10.3897/BDJ.14.e191556 (PMC13379711; doi:10.3897/BDJ.14.e191556)

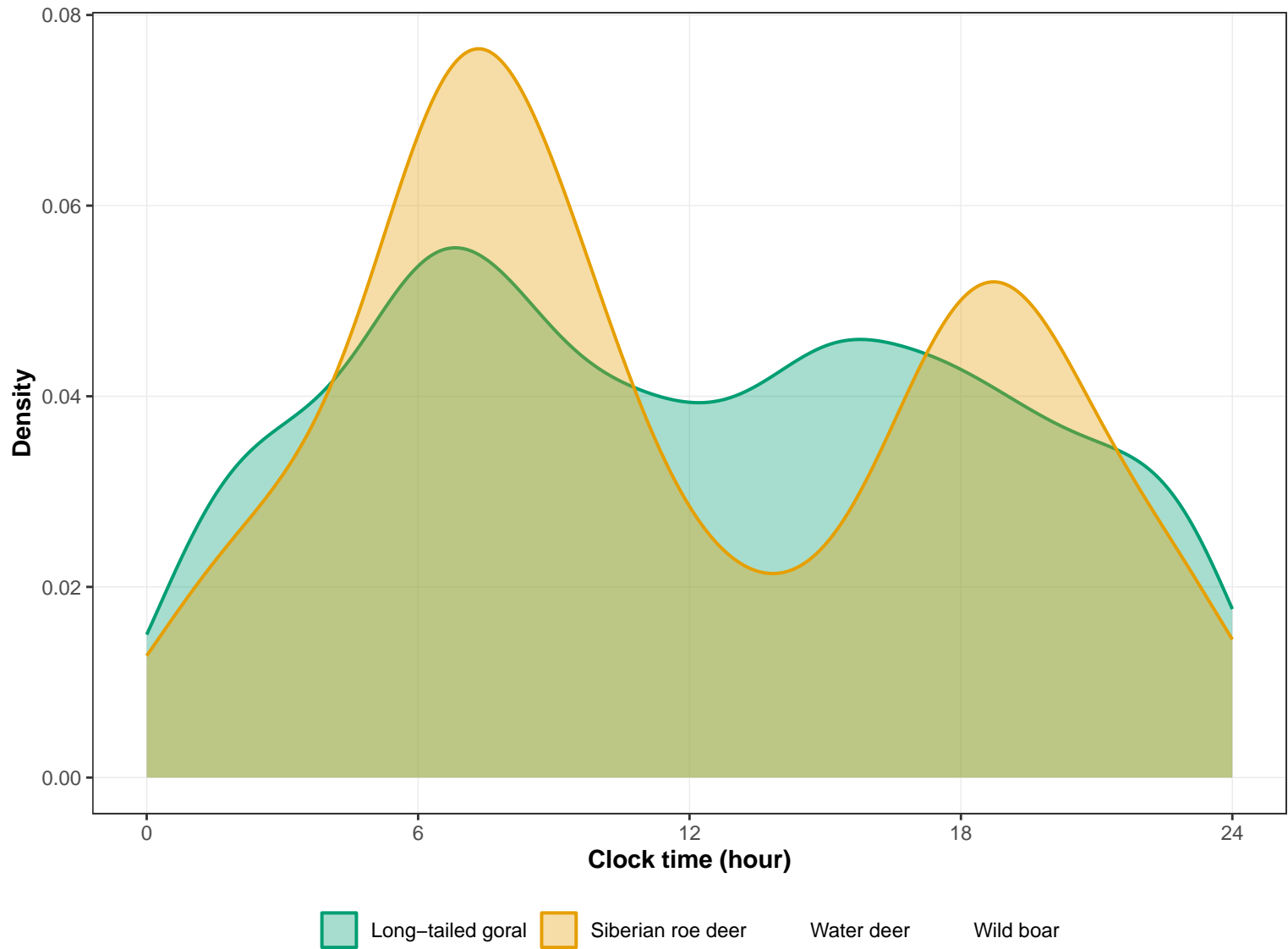

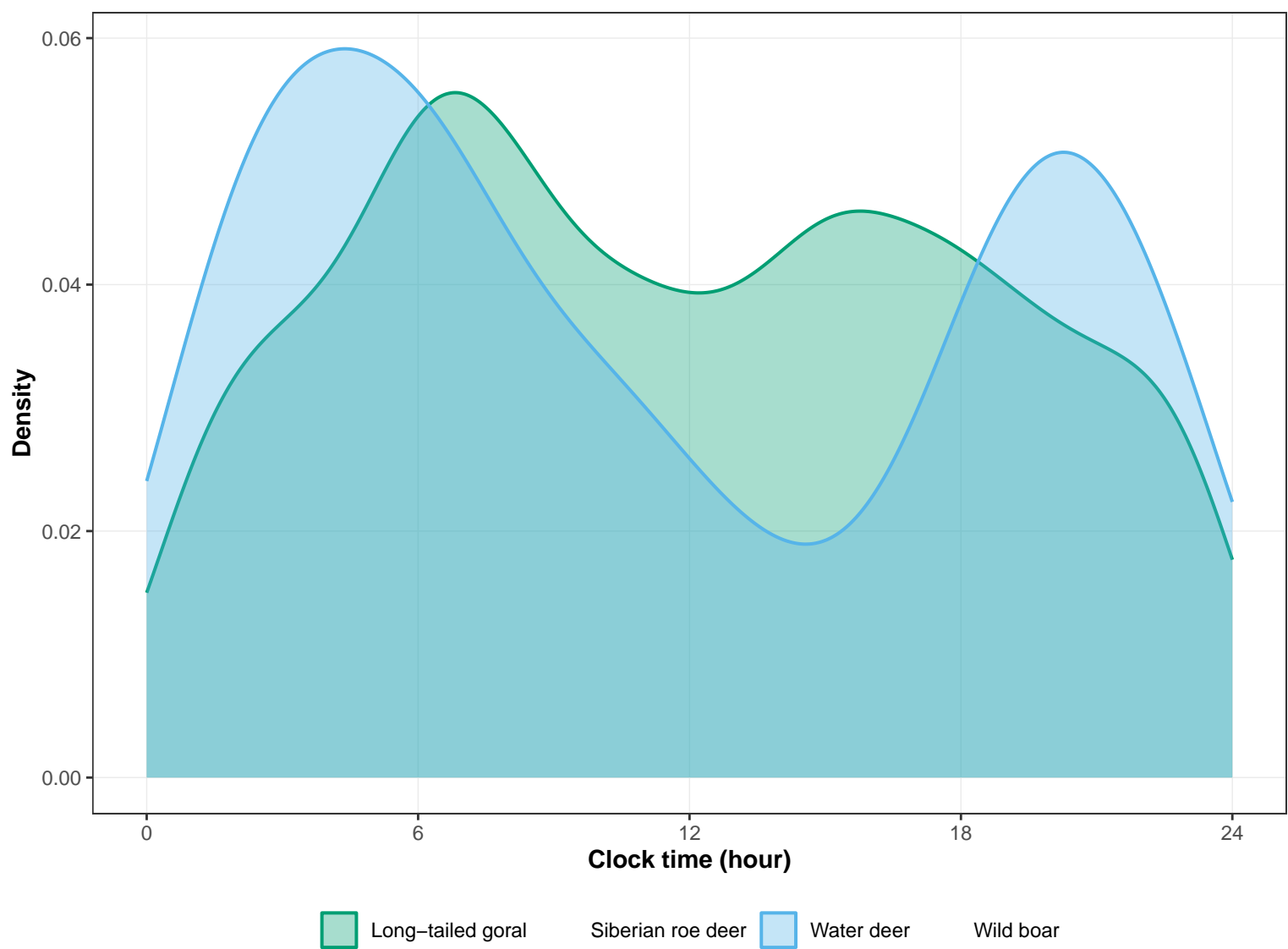

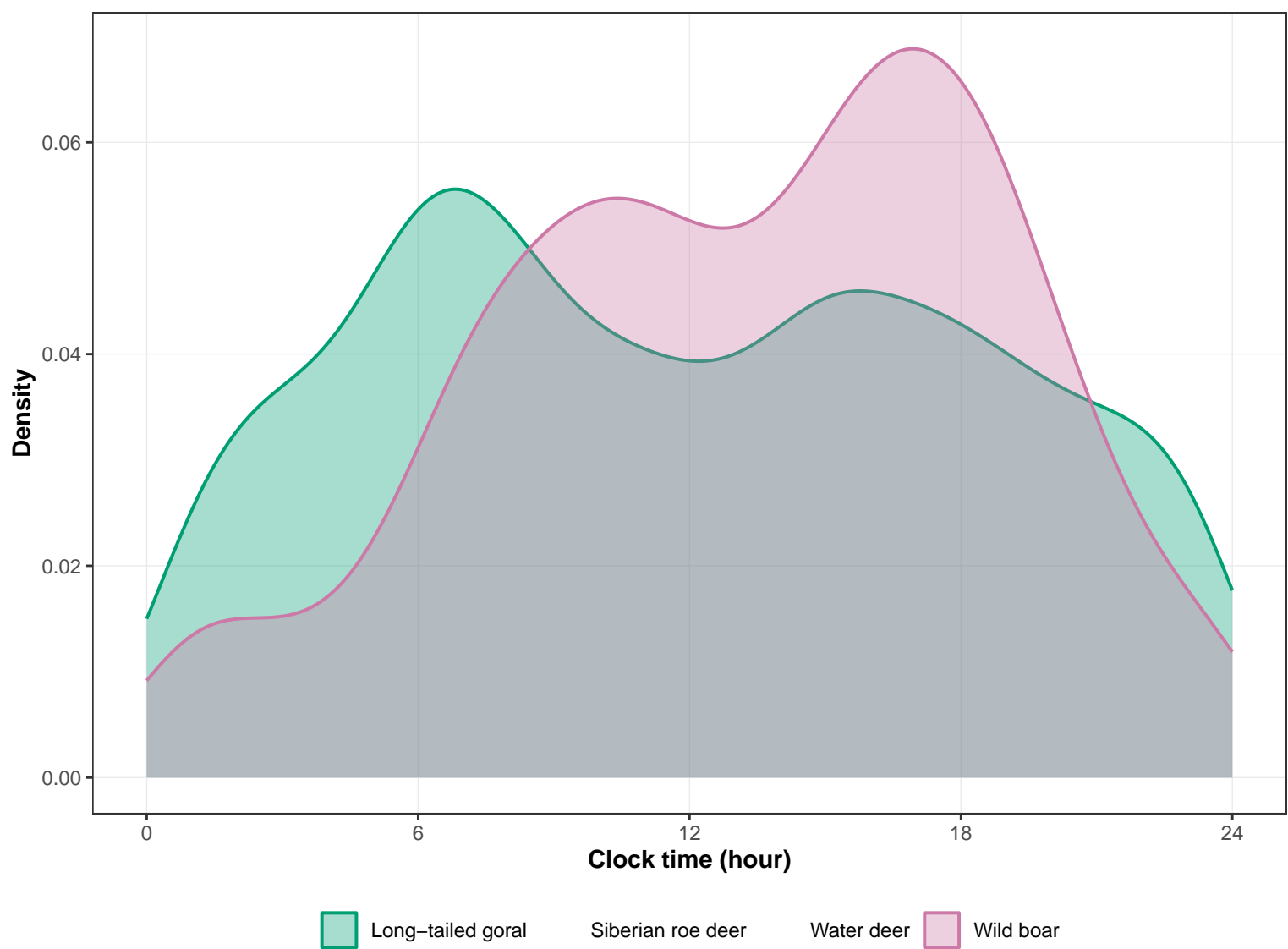

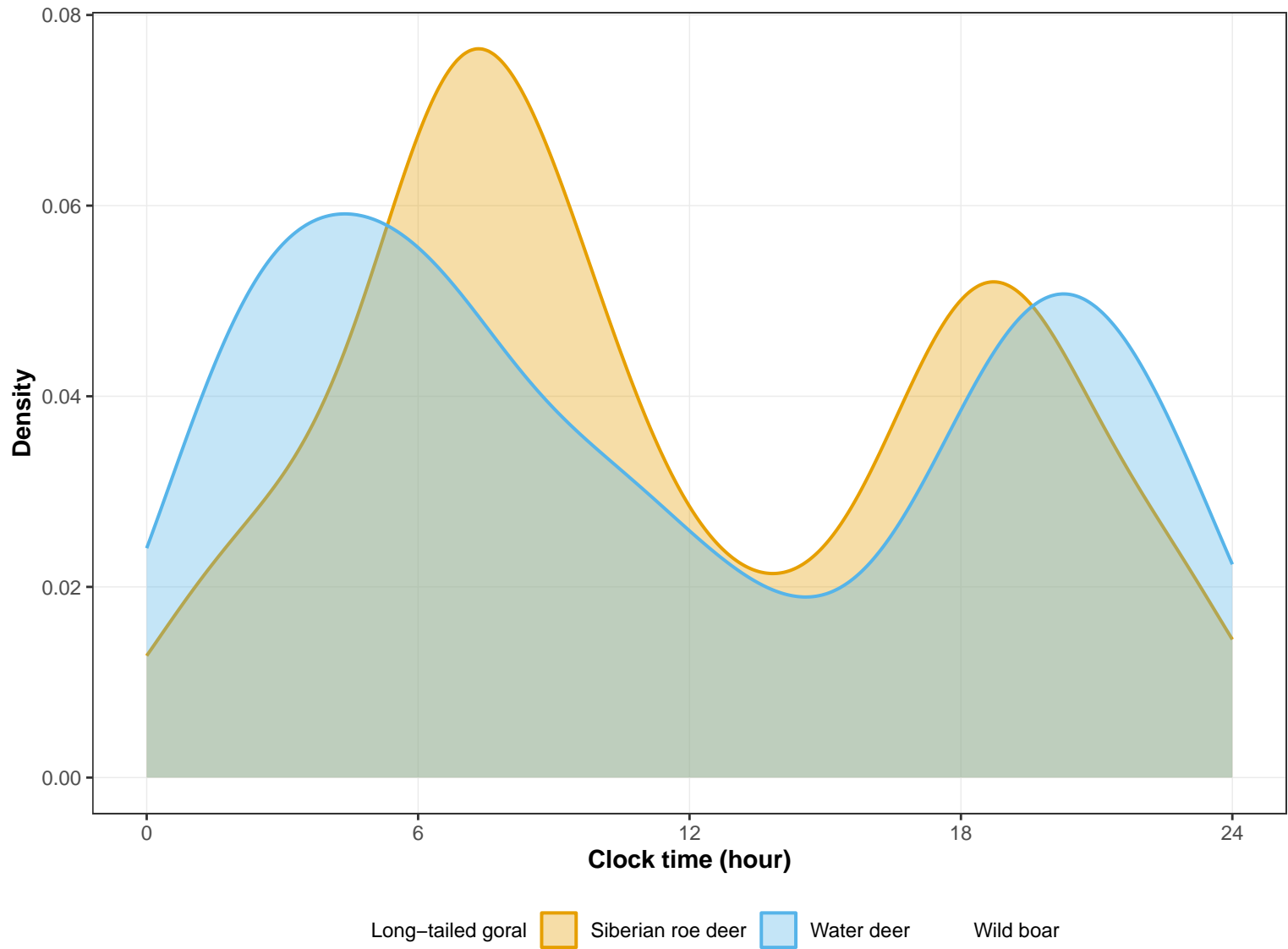

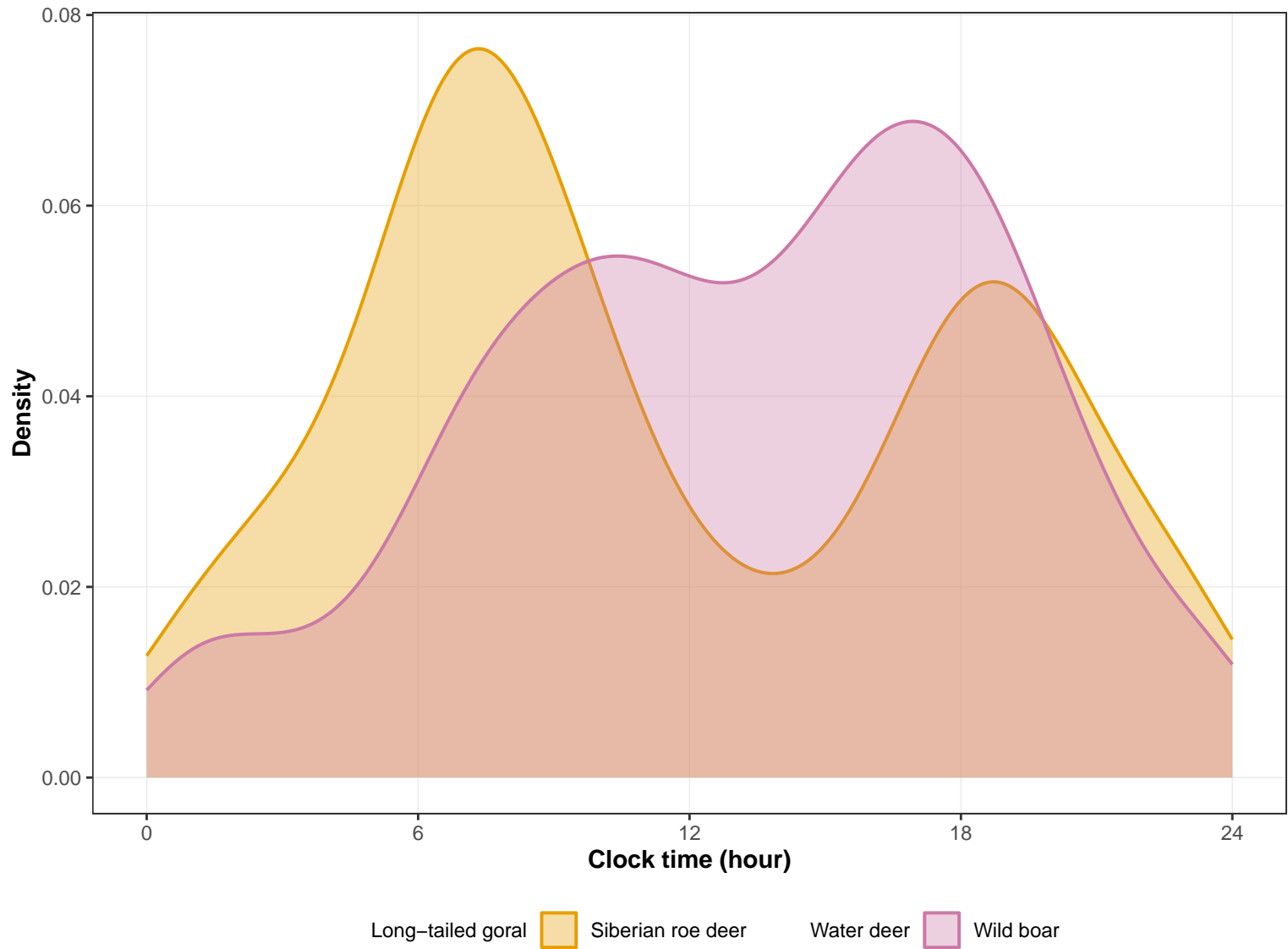

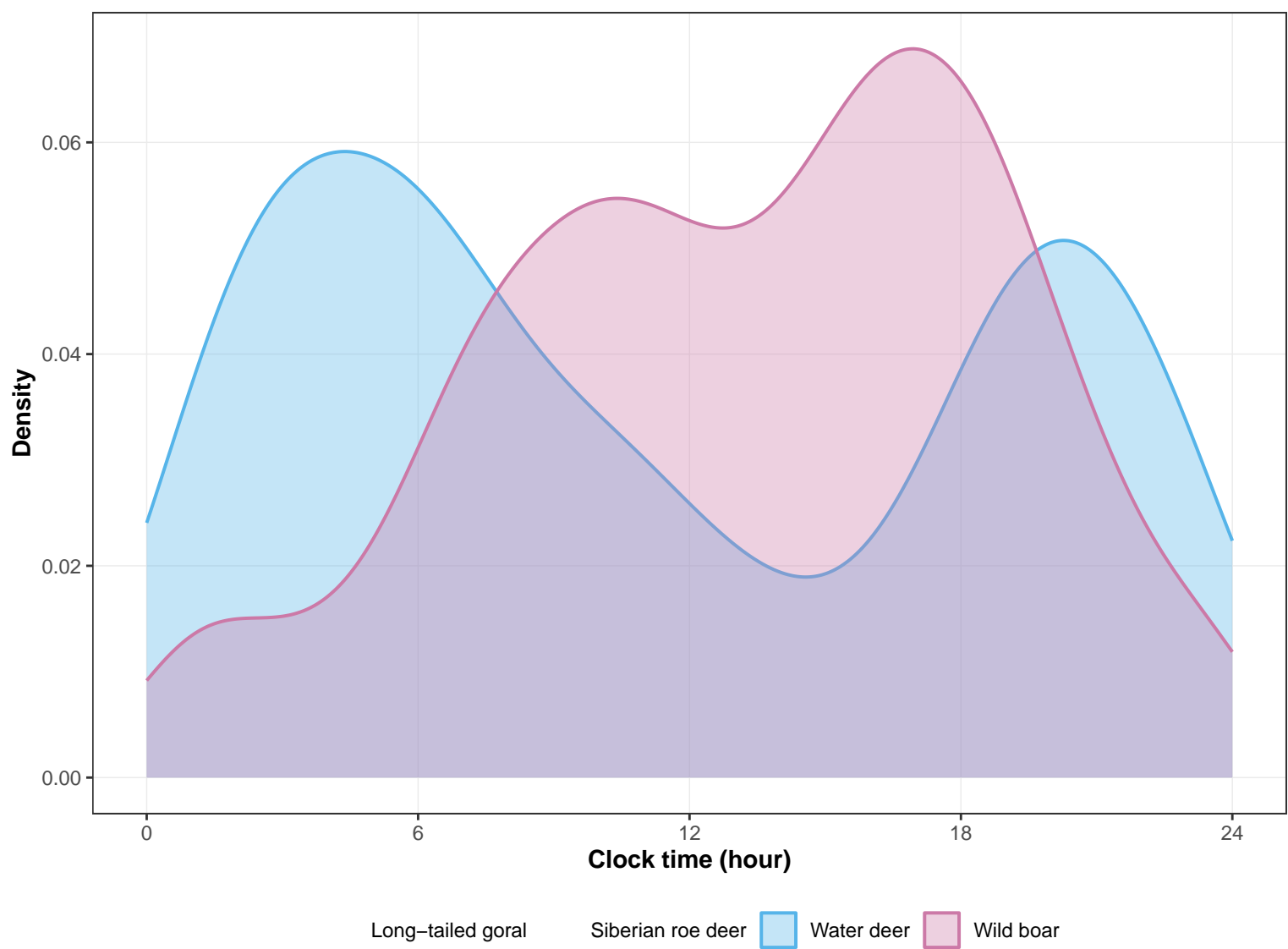

Supplement: Supplementary material 1 — Pairwise density plots for all species pairs [file bdj-14-e191556-s001.zip › SupFig_Overlap_PairwiseDensityPlots.pdf]

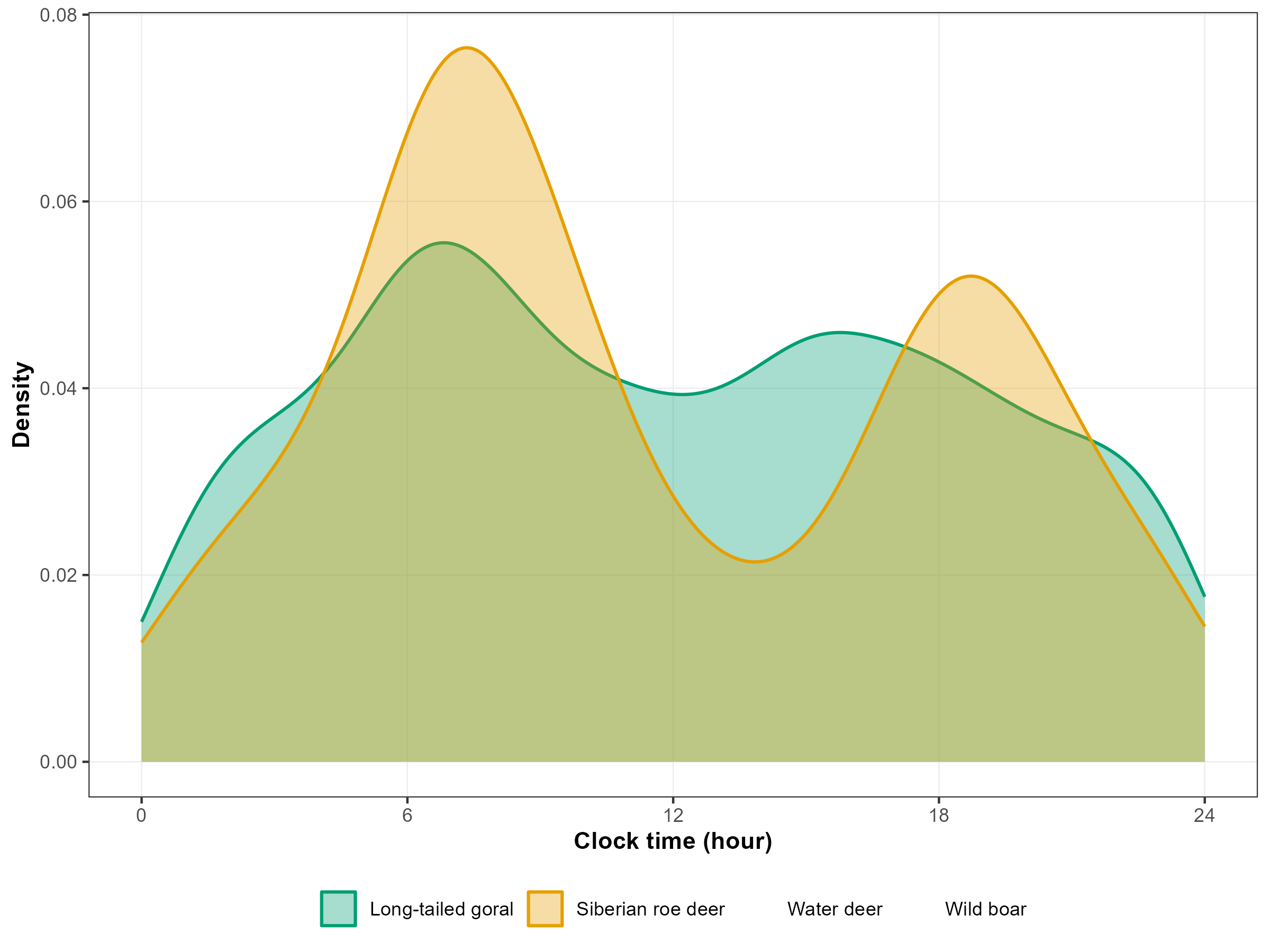

Supplement: Supplementary material 1 — Pairwise density plots for all species pairs [file bdj-14-e191556-s001.zip › SupFig_pairs_png/SupFig_pair_Long-tailed_goral_vs_Siberian_roe_deer.png]

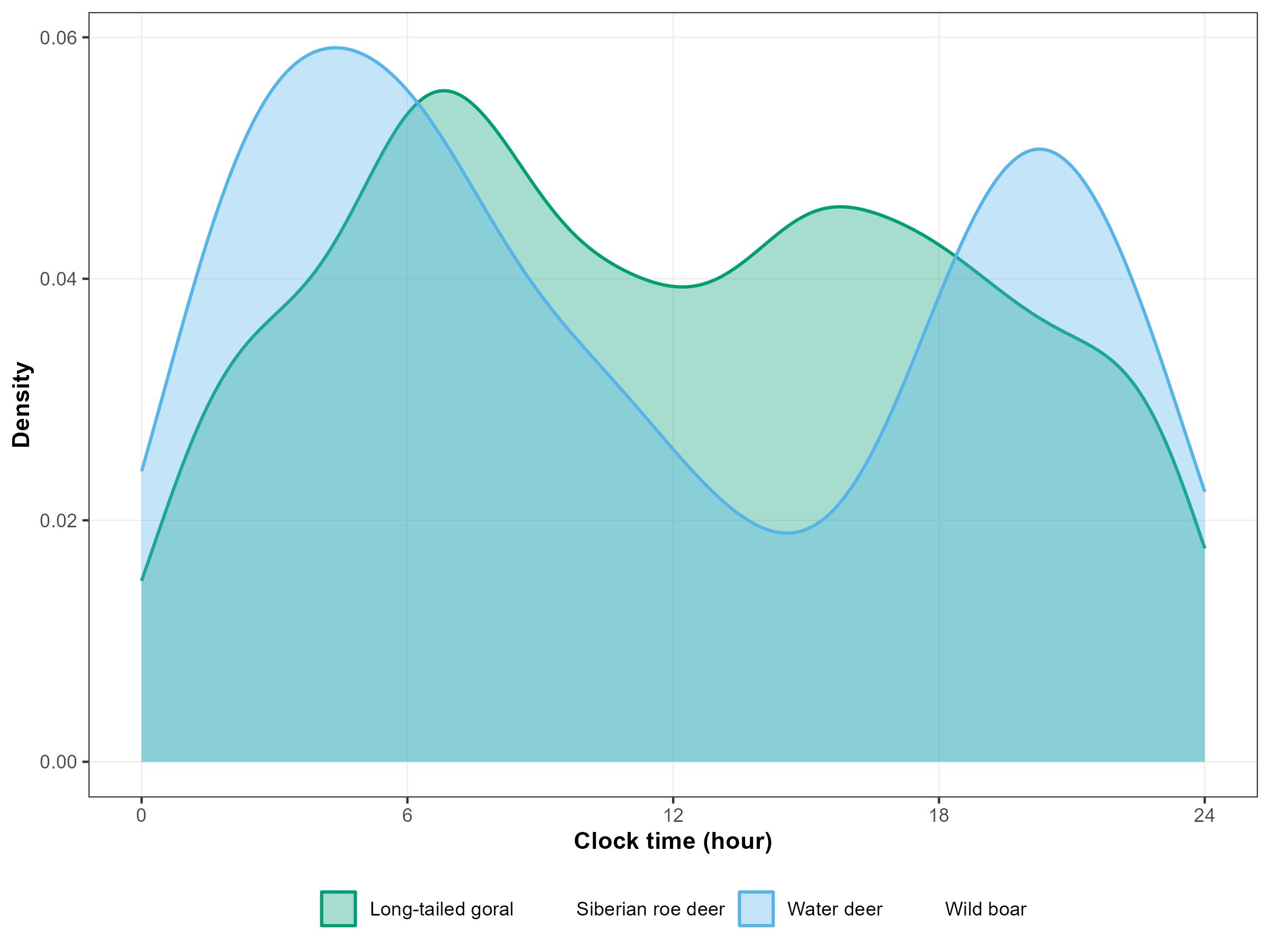

Supplement: Supplementary material 1 — Pairwise density plots for all species pairs [file bdj-14-e191556-s001.zip › SupFig_pairs_png/SupFig_pair_Long-tailed_goral_vs_Water_deer.png]

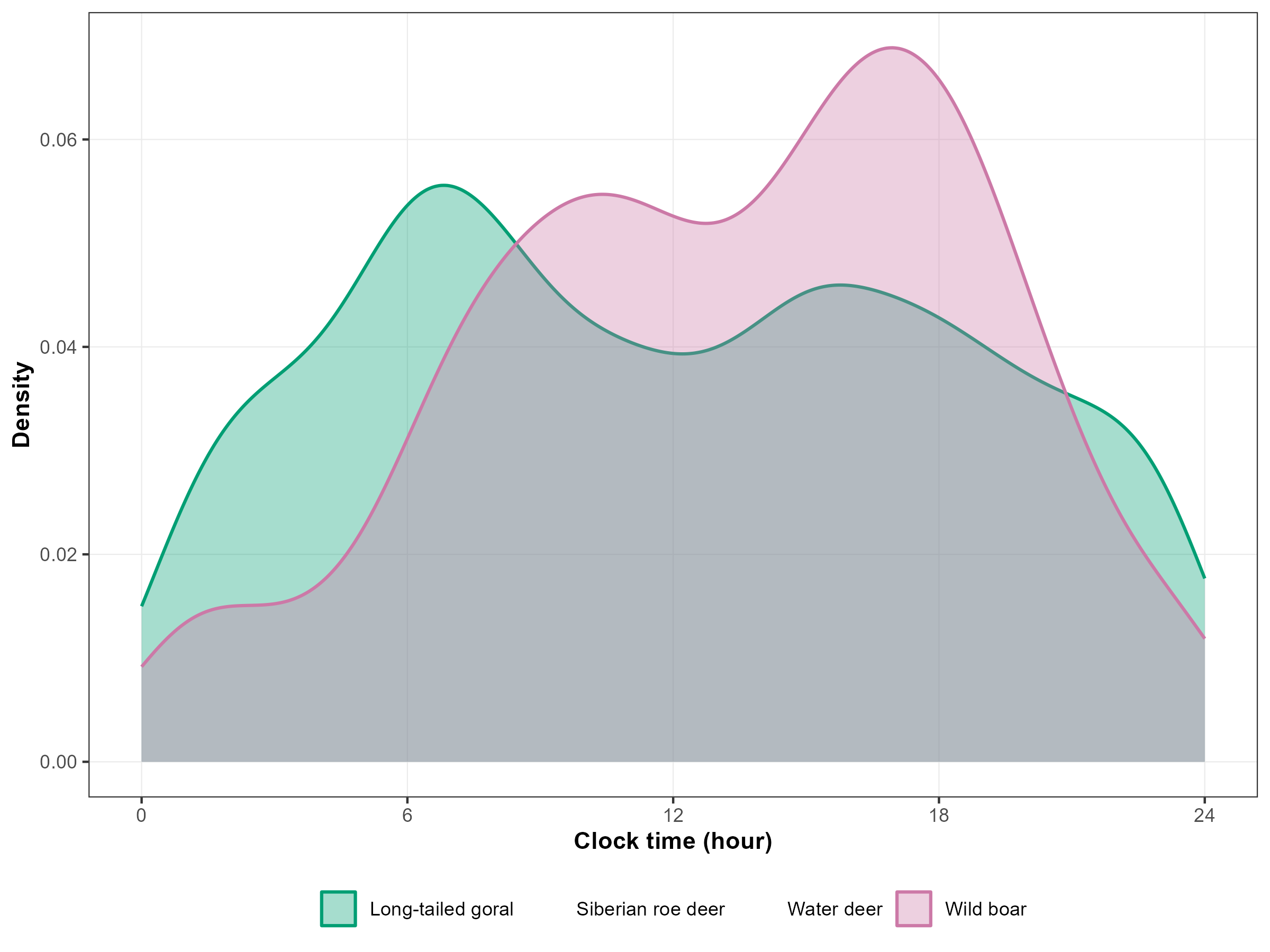

Supplement: Supplementary material 1 — Pairwise density plots for all species pairs [file bdj-14-e191556-s001.zip › SupFig_pairs_png/SupFig_pair_Long-tailed_goral_vs_Wild_boar.png]

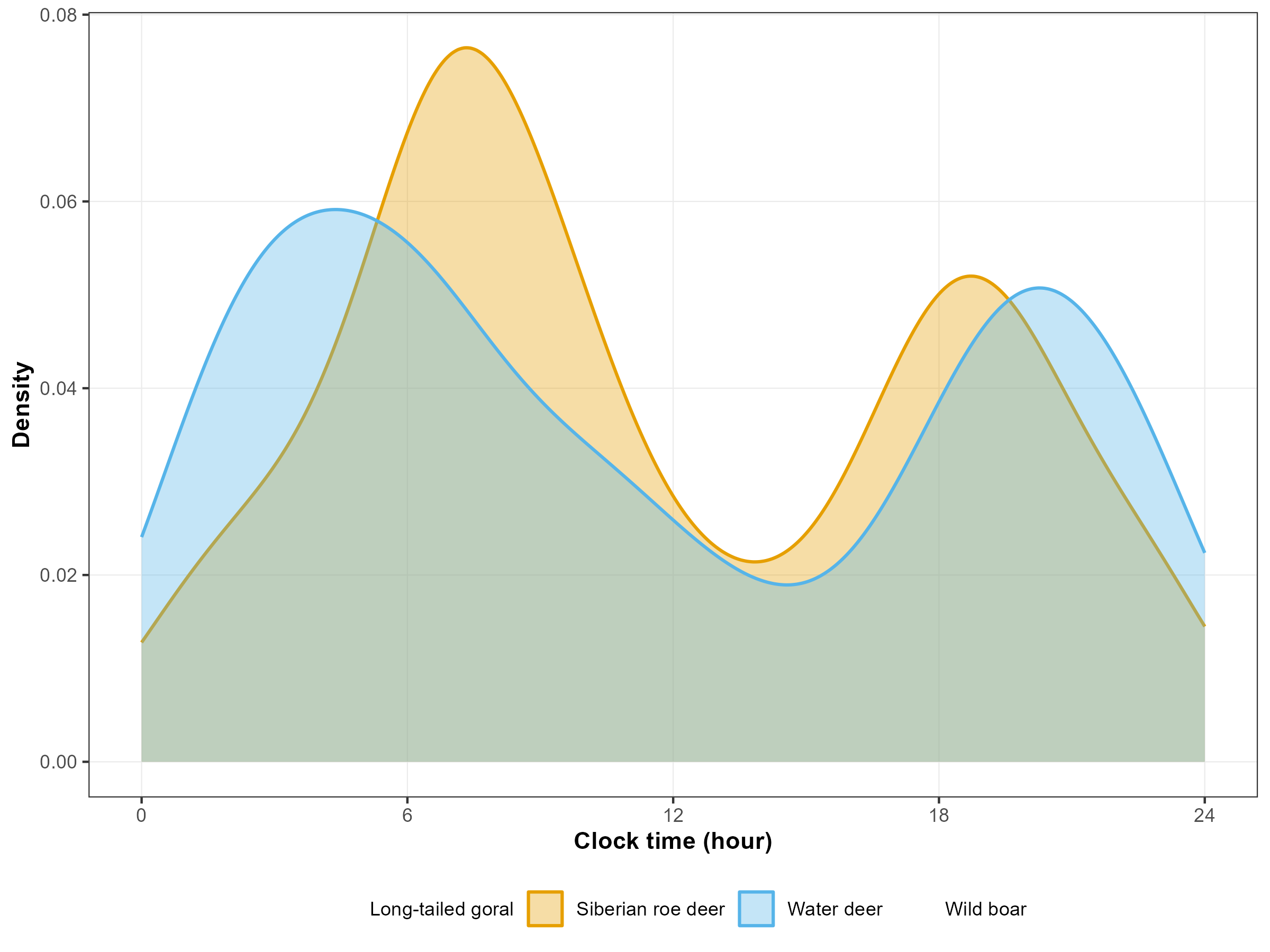

Supplement: Supplementary material 1 — Pairwise density plots for all species pairs [file bdj-14-e191556-s001.zip › SupFig_pairs_png/SupFig_pair_Siberian_roe_deer_vs_Water_deer.png]

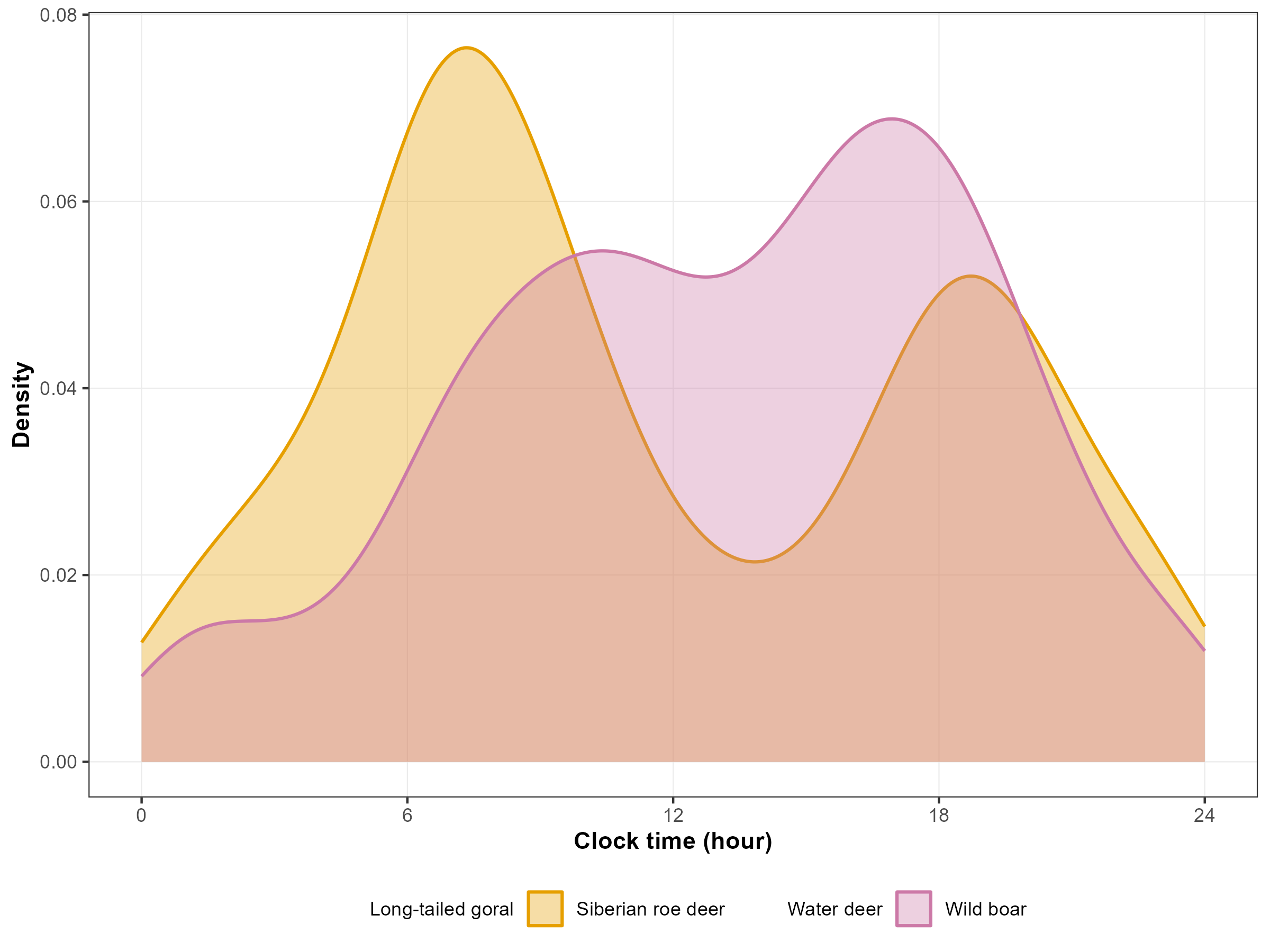

Supplement: Supplementary material 1 — Pairwise density plots for all species pairs [file bdj-14-e191556-s001.zip › SupFig_pairs_png/SupFig_pair_Siberian_roe_deer_vs_Wild_boar.png]

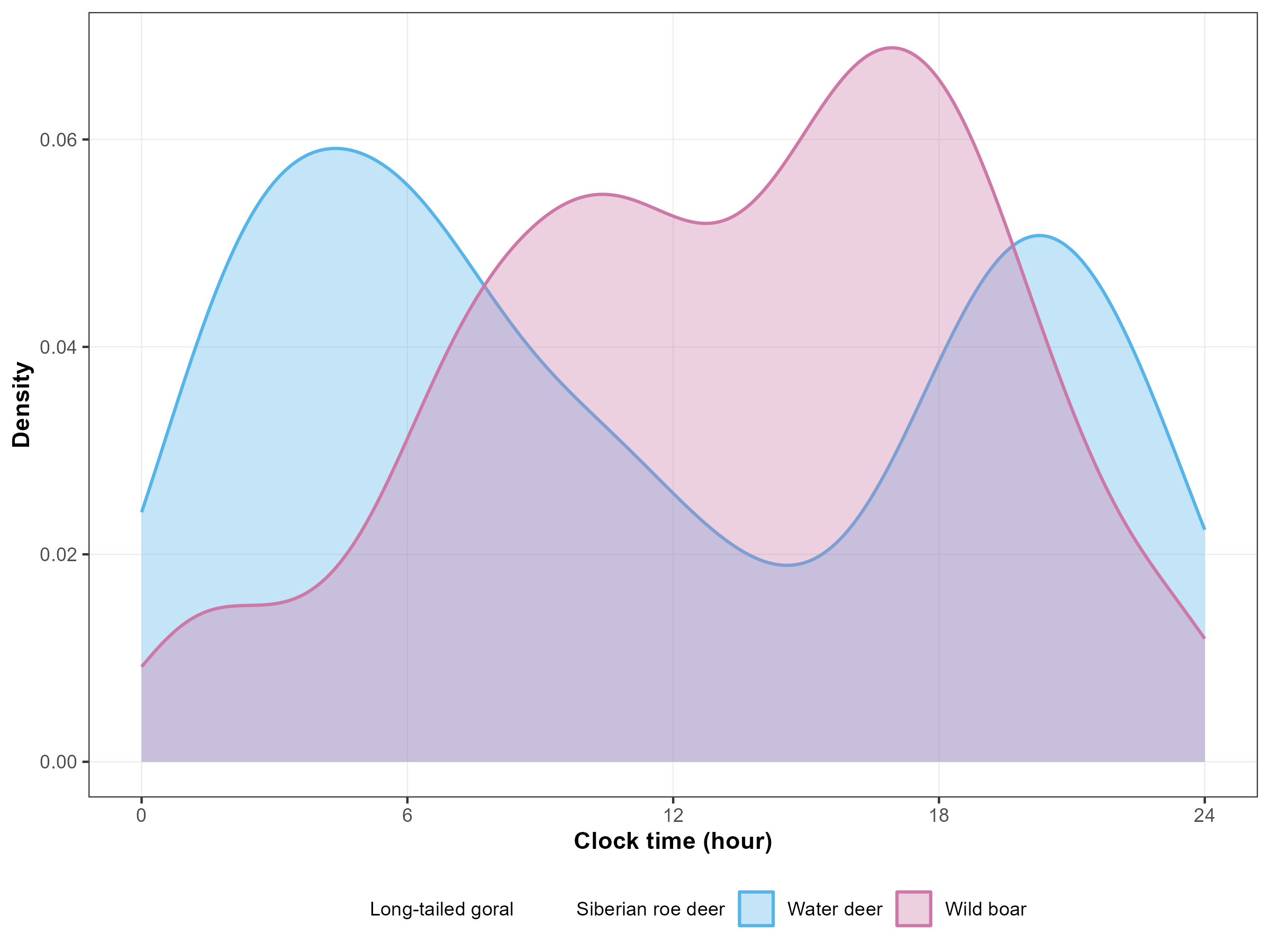

Supplement: Supplementary material 1 — Pairwise density plots for all species pairs [file bdj-14-e191556-s001.zip › SupFig_pairs_png/SupFig_pair_Water_deer_vs_Wild_boar.png]

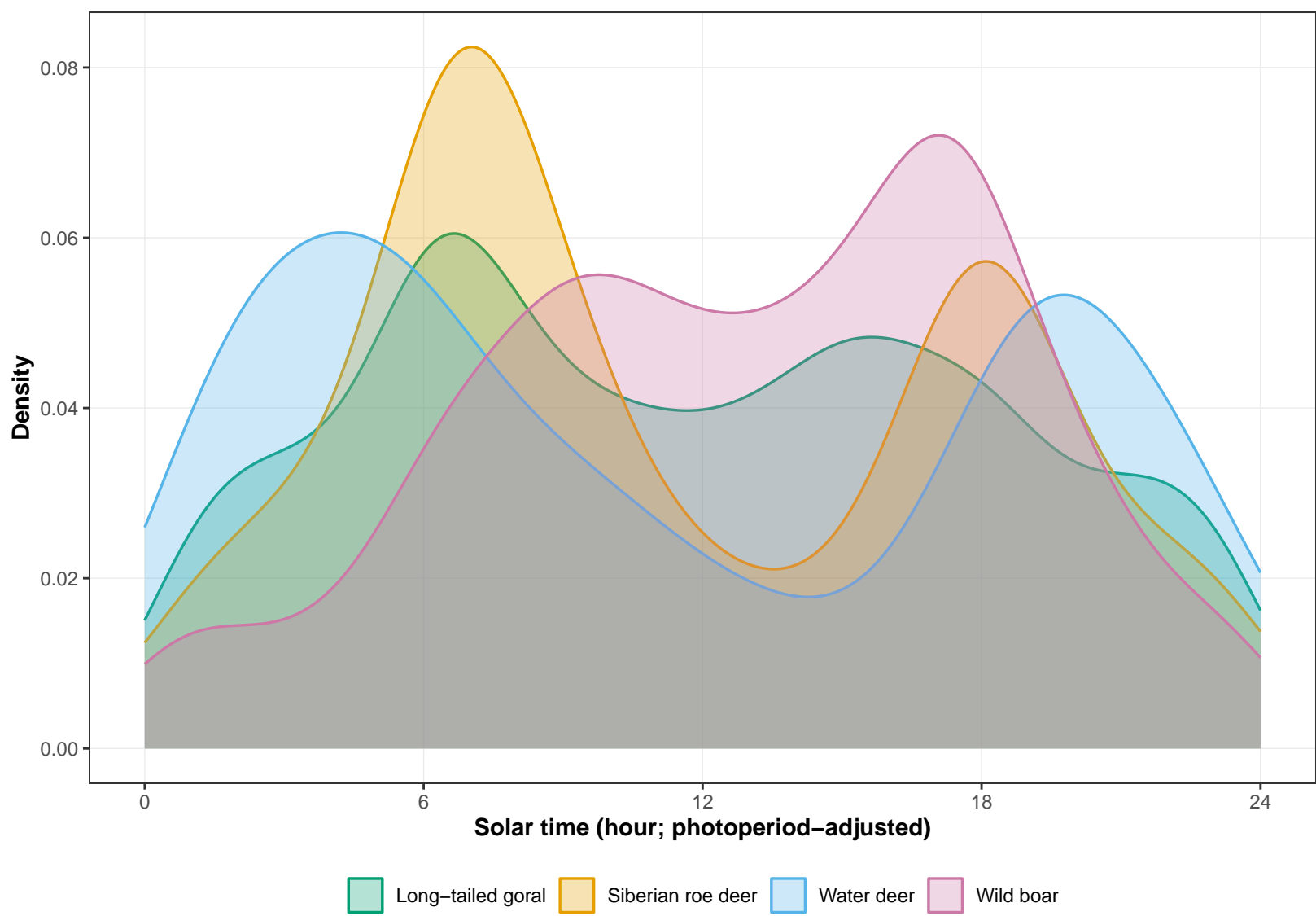

Supplement: Supplementary material 2 — Photoperiod-adjusted (solar-time) activity curves and overlap estimates [file bdj-14-e191556-s002.zip › EA02_Solar_Fig_ActivityDensity_bySpecies_SolarTime.pdf]

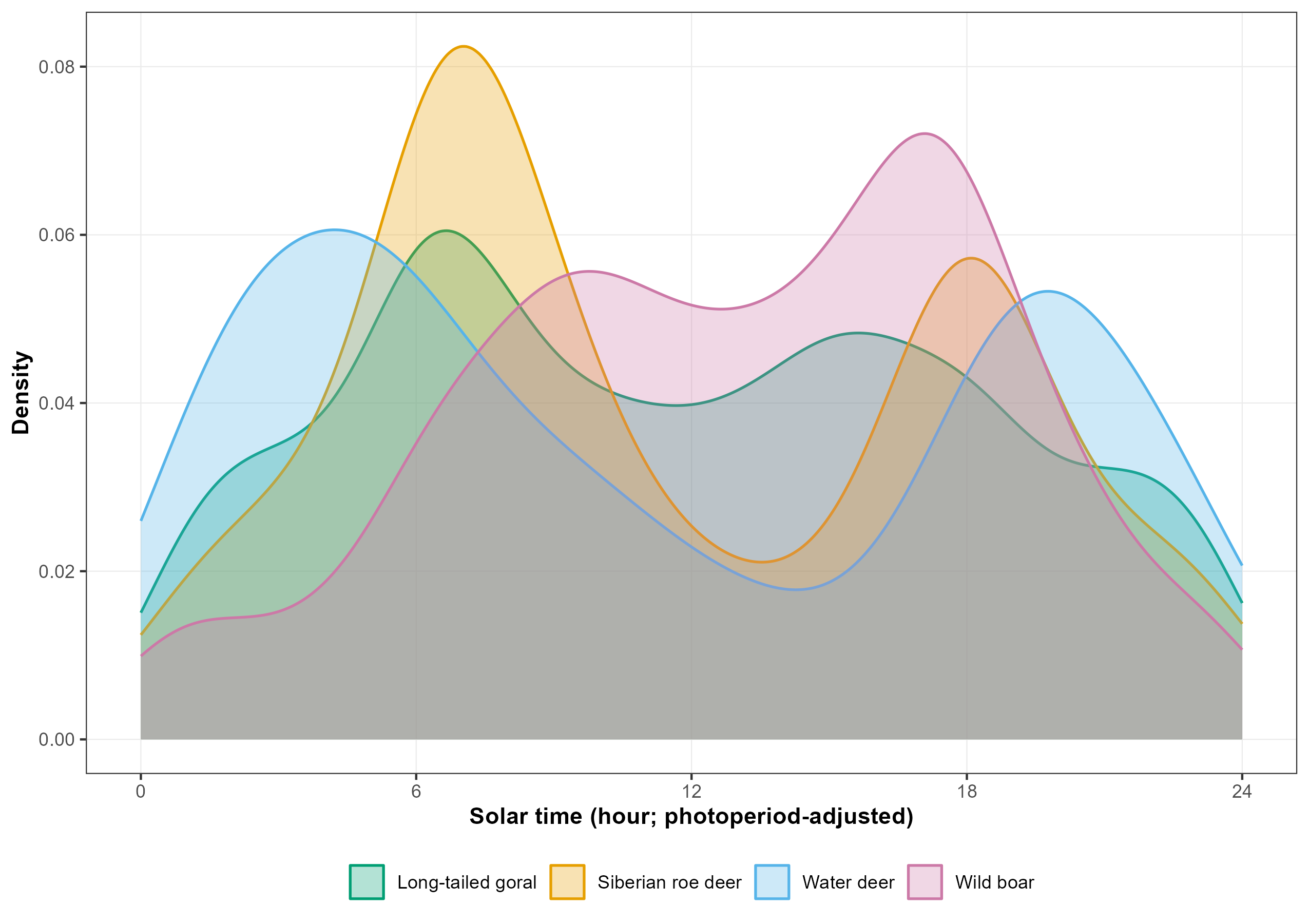

Supplement: Supplementary material 2 — Photoperiod-adjusted (solar-time) activity curves and overlap estimates [file bdj-14-e191556-s002.zip › EA02_Solar_Fig_ActivityDensity_bySpecies_SolarTime.png]

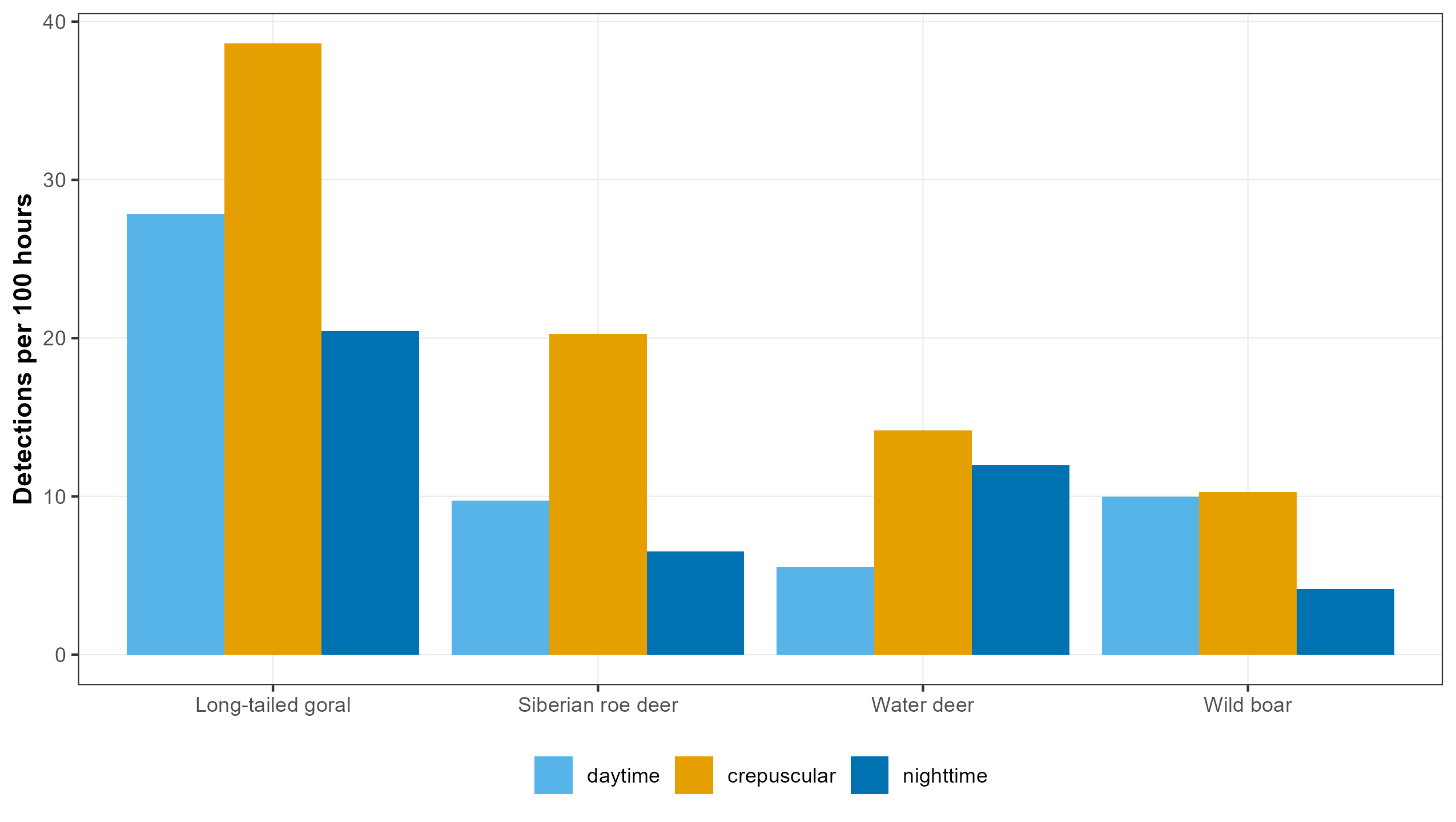

Supplement: Supplementary material 3 — Diel-period rate-normalised summaries (detections per 100 hours) [file bdj-14-e191556-s003.zip › EA02_DielRate_Fig_RatePer100h.png]
